# Gut-Microbiota-Metabolite Axis in Early Renal Function Decline

**DOI:** 10.1371/journal.pone.0134311

**Published:** 2015-08-04

**Authors:** Clara Barrios, Michelle Beaumont, Tess Pallister, Judith Villar, Julia K. Goodrich, Andrew Clark, Julio Pascual, Ruth E. Ley, Tim D. Spector, Jordana T. Bell, Cristina Menni

**Affiliations:** 1 Department of Twin Research and Genetic Epidemiology, King’s College London, London, United Kingdom; 2 Department of Nephrology, Hospital del Mar, Institut Mar d’Investigacions Mediques, Barcelona, Spain; 3 Department of Infectious Diseases, Hospital del Mar, Institut Mar d’Investigacions Mediques, Barcelona, Spain; 4 Department of Molecular Biology and Genetics, Cornell University, Ithaca, United States of America; Mario Negri Institute for Pharmacological Research and Azienda Ospedaliera Ospedali Riuniti di Bergamo, ITALY

## Abstract

**Introduction:**

Several circulating metabolites derived from bacterial protein fermentation have been found to be inversely associated with renal function but the timing and disease severity is unclear. The aim of this study is to explore the relationship between indoxyl-sulfate, p-cresyl-sulfate, phenylacetylglutamine and gut-microbial profiles in early renal function decline.

**Results:**

Indoxyl-sulfate (Beta(SE) = -2.74(0.24); *P* = 8.8x10^-29^), p-cresyl-sulfate (-1.99(0.24), *P* = 4.6x10^-16^), and phenylacetylglutamine(-2.73 (0.25), *P* = 1.2x10^-25^) were inversely associated with eGFR in a large population base cohort (TwinsUK, n = 4439) with minimal renal function decline. In a sub-sample of 855 individuals, we analysed metabolite associations with 16S gut microbiome profiles (909 profiles, QIIME 1.7.0). Three Operational Taxonomic Units (OTUs) were significantly associated with indoxyl-sulfate and 52 with phenylacetylglutamine after multiple testing; while one OTU was nominally associated with p-cresyl sulfate. All 56 microbial members belong to the order Clostridiales and are represented by anaerobic Gram-positive families Christensenellaceae, Ruminococcaceae and Lachnospiraceae. Within these, three microbes were also associated with eGFR.

**Conclusions:**

Our data suggest that indoxyl-sulfate, p-cresyl-sulfate and phenylacetylglutamine are early markers of renal function decline. Changes in the intestinal flora associated with these metabolites are detectable in early kidney disease. Future efforts should dissect this relationship to improve early diagnostics and therapeutics strategies.

## Introduction

It is increasingly recognized that the microbiome may affect health and disease of the host. Indeed the endogenous flora has been recently associated with type 2 diabetes, obesity, metabolic syndrome, cancer and liver cirrhosis among others [1–4]

Metabolites derived from bacteria provide a readout of the metabolic state of an individual and are the product of genetic [5,6] and exogenous (diet, lifestyle, gut microbial activity) factors under a particular set of conditions [7]. Under physiological conditions, there is a balance between the intestinal bacteria and the host, due to the innate immunity that maintains equilibrium in inflammation pathways and the intestinal barrier integrity. However, in chronic kidney disease (CKD), the uremic environment affects the intestinal barrier leading to bacterial dysbiosis [8]. This activates inflammatory pathways and immune processes and leads to systemic inflammation [9]. However, the degree of renal impairment that leads into modification of the intestinal milieu or the deficit of gut-metabolites excretion remains unclear.

A deeper understanding of the gut-microbe-metabolite axis is a prerequisite to improve therapeutic strategies that manipulate the gut microbiota in the onset of kidney dysfunction. Indoxyl-sulfate and p-cresyl-sulfate are end-products of bacterial protein fermentation of tryptophan and tyrosine respectively in the colon [10]. In vitro and ex vivo data show that indoxyl-sulfate and p-cresyl-sulfate may trigger or accelerate cardiovascular disease and progression of kidney failure [11,12]. Clinical observational studies also correlate high levels of both metabolites with overall mortality as well as cardiovascular disease and renal disease progression [13–15]. Phenylacetylglutamine is a major nitrogenous metabolite that accumulates in uremia. Its plasma levels increase after cigarette smoke exposure, in ischemic heart failure patients, hypertension, cardiovascular risk [16] and in the progression to end stage renal disease in type2 diabetic patients [17–19].

To date studies have concentrated on changes in intestinal flora and gut-metabolite levels in advanced stages of CKD [8,9,15,20–24], but potential changes in intestinal microbiota and gut microbial metabolites in early renal function decline have not yet been fully explored. To this end, we analyzed the links between metabolites indoxyl-sulfate, p-cresyl-sulfate and phenylacetylglutamine and gut microbiota to investigate whether changes at the individual operational taxonomic units (OTUs) level are detectable in early renal function decline.

## Results and Discussion

Association of plasma circulating metabolites derived from bacterial protein fermentation was analyzed in 4439 individuals with different eGFR from the TwinsUK cohort. The demographic characteristics of the study populations are presented in **Table 1**. Out of 4439 individuals only 7.4% had eGFR<60 mL/min/1.73m^2^. Indoxyl-sulfate (Beta(SE) = -2.74(0.24), *P* = 8.8x10^-29^), p-cresyl-sulfate (-1.99(0.24), *P* = 4.6x10^-16^), and phenylacetylglutamine (-2.73(0.25), *P* = 1.2x10^-25^) were significantly and negatively associated with eGFR after adjusting for age, sex, body mass index (BMI), metabolite batch, type 2 diabetes, family relatedness and multiple testing using Bonferroni correction (**Table 2**).

**Table 1:** General Characteristics of the study population. Left column: Characteristics of population with renal and plasma metabolites data analyzed. Right column: Characteristics of sub-population with faecal microbiota data analyzed.

	Metabolites	Microbiota
**Sample size, *n***	4439	855
**Age, *yrs***	53.04±14.08	58.39±10.88
**MZ:DZ:singletons**	1795:1980:664	288:414:152
**Female, *n (%)***	4162 (93.7)	840 (98.2)
**BMI, *Kg/m*** ^***2***^	25.94±4.79	26.14±4.77
**Creatinine, *mg/mL***	0.83±0.25	0.80±0.16
**eGFR, *mL/min/1*.*73m*** ^***2***^	84.93±16.85	83.06±15.42
**CKD (eGFR ≤ 60), *n (%)***	331 (7.4)	62 (7.2)
**Type2 Diabetes, *n (%)***	78 (1.7)	21 (2.4)

CKD = Chronic Kidney Disease. eGFR = estimated glomerular filtration rate (CKD-EPI equation). MZ = monozygotic, DZ = dizygotic. Values for categorical variables are given as n (percentage); values for continuous variable as mean (± SD).

**Table 2:** Association and correlation of the metabolites and the eGFR.

Metabolites	eGFR		*h* ^*2*^ *[95%CI]* *
	Beta(SE)	p	
Indoxyl-sulphate	-2.74 (0.24)	8.8x10^-29^	0.24[0.12;0.37]
p-cresyl-sulphate	-1.99 (0.24)	4.6x10^-16^	0.36[0.28;0.40]
Phenylacetylglutamine	-2.73 (0.25)	1.2x10^-25^	0.33[0.21;0.44]

eGFR = estimated glomerular filtration rate. *h* ^*2*^ = *Heritability*.

*heritability estimates come from *Shin SY et al Nat Genet 2014 [6].*

As dietary factors are known to affect metabolites to varying levels [25,26], we tested their effect on the association between the metabolites and eGFR by including them as covariates in the linear model. Results were unchanged suggesting that dietary factors do not confound the three metabolite-eGFR association.

The plasma levels of these metabolites reflect the balance between elimination and generation. Some studies suggest most of the microbial derived metabolites are protein-bound [27], hence, elimination would depend on eGFR and the tubular transporter system.

A recent study, showed that eGFR provides an acceptable estimate of renal clearance of indoxyl and p-cresyl sulfate (*R* ^*2*^ = *0*.*75*, *p<0*.*001*) in subjects with eGFR < 60mL/min/1.73m^2^ [28]. These metabolites may be more sensitive to earlier stages of reduced renal function, as the eGFR-defined onset of CKD occurs only after half of the kidneys’ filtration ability has been lost. Moreover, its higher levels in blood suggest the environmental changes affecting the intestinal flora could be playing a role in modifying the intestinal barrier before the onset of CKD.

**Fig 1:**
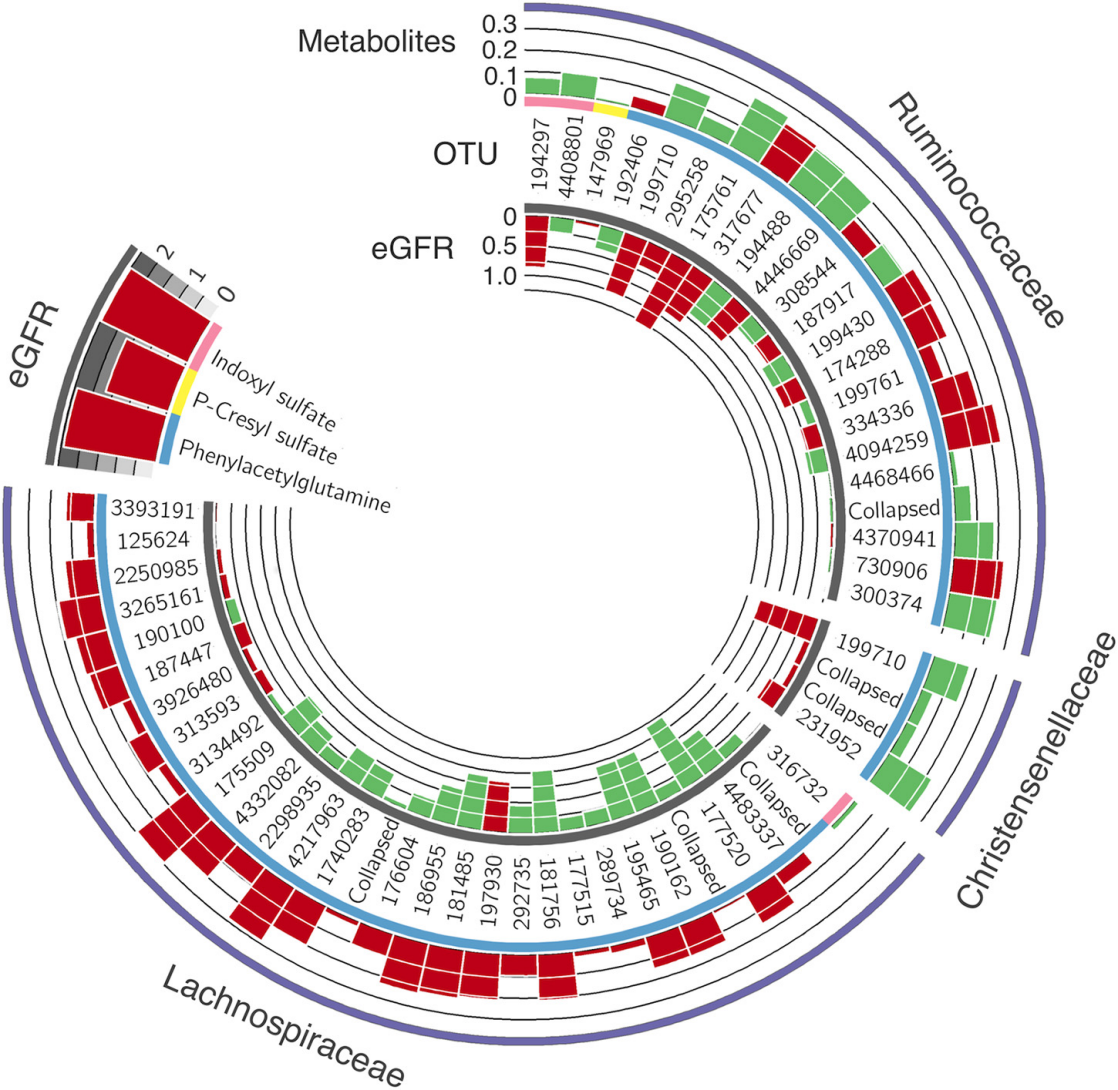
Circus histogram depicts positive and negative associations between the operational taxonomic units (OTUs) (middle circle), the metabolites (external circle) and the glomerular filtration rate (eGFR) (internal circle). Green color shows positive association while red color shows negative. The heights of the histogram columns represent the beta coefficients in the regression model. Upper left histograms represent the beta coefficients for the association between the three plasma metabolites and the eGFR. All the microbial traits belong to the order of Clostridiales and are represented by the families: Christensenellaceae, Ruminococcaceae and Lachnospiraceae.

We used 16S gut microbiome data available in a subset of the TwinsUK cohort individuals, to test for association between eGFR and plasma levels of indoxyl sulfate, p-cresyl sulfate and phenylacetylglutamine with 909 gut-microbial profiles (768 Operational Taxonomic Units (OTUs) and 141 collapsed taxonomies; see Methods). The gut microbiome 16s data have previously been described [29] and the current study analyzed a subset of 855 individuals with microbiome, fasting blood metabolites and eGFR data available (see demographic characteristics of the study population in **Table 1**). After adjusting for age, sex, BMI, metabolite batch, family relatedness and controlling for multiple testing using false discovery rate (FDR <5%), 3 OTUs were significantly associated with indoxyl-sulphate and 52 with phenylacetylglutamine (see **Fig 1** and **Table 3** for the full list). One OTU showed a borderline significance association with p-cresyl-sulphate but did not reach the FDR threshold. All the 56 microbial profiles belong to the order of Clostridiales and are mainly represented by the anaerobic Gram-positive families: Christensenellaceae, Ruminococcaceae and Lachnospiraceae. We then tested for association between these 56 microbies and renal function. After adjusting for covariates, 3 microbes were nominally associated with eGFR, and 2 were among those associated with phenylacetylglutamine and one with indoxyl-sulphate (see **Fig 1** and **Table 3** for the full list). Microbes can also be affected by diet [29] and antibiotic use [30] and we therefore rerun the analyses adjusting for these confounders. Results were in line with those from the overall cohort, suggesting dietary pattern and antibiotic used are not affecting our associations. However, as data on diet and antibiotics was available for only 11% of the subjects with microbiota data, we cannot draw a more robust conclusion.

**Table 3:** Operational taxonomic units (OTUs) belonging to the order Clostridiales and represented by Ruminococcaceae, Christensenellaceae and Lachnospiraceae families. Table shows those significantly associated with Indoxyl sulfate and phenylacetylglutamine, the nominally associated with p-Cresyl sulfate and its association with the eGFR.

	**Gut Microbes**			***h*** ^***2***^ ***[95%CI]*** *	**Metabolites**			**eGFR**	
	**Taxonomy**				**Indoxyl-Sulfate**				
**OTU**	**Order**	**Family**	**Genus.species**		**Beta(SE)**	**p**	**qval** **	**Beta(SE)**	**p**
194297	Clostridiales	Ruminococcaceae	Ruminococcus	0.13 [0;0.25]	0.09(0.02)	0.0002	0.020	-0.86(0.44)	0.049
316732	Clostridiales	Lachnospiraceae	Lachnospira	0.2 [0;0.34]	0.04(0.01)	0.0006	0.036	0.02(0.20)	0.092
4408801	Clostridiales	Ruminococcaceae	Oscillospira	0.23 [0;0.35]	0.12(0.03)	0.0007	0.038	0.25(0.54)	0.063
					**Metabolites**			**eGFR**	
					**p-Cresyl**				
					**Beta(SE)**	**p**	**qval** **	**Beta(SE)**	**p**
147969	Clostridiales	Ruminococcaceae	Ruminococcus	0 [0;0.0]	0.03(0.009)	0.002	0.057	-0.06(0.22)	0.077
					**Metabolites**			**eGFR**	
					**Phenylacetylglutamine**				
					**Beta(SE)**	**p**	**qval** **	**Beta(SE)**	**p**
Collapsed	Clostridiales	Lachnospiraceae	Unclassified	0.21 [0;0.35]	-0.02(0.06)	0.0011	0.047	0.21(0.09)	0.028
4483337	Clostridiales	Lachnospiraceae	Unclassified	0 [0;0.3]	-0.08(0.02)	0.0002	0.021	0.67(0.33)	0.043
177520	Clostridiales	Lachnospiraceae	Roseburia faecis	0 [0;0.0]	-0.17(0.04)	0.0002	0.021	1.21(0.63)	0.056
192406	Clostridiales	Ruminococcaceae	Unclassified	0.12 [0;0.24]	-0.07(0.01)	2.92x10^-5^	0.009	0.40(0.26)	0.133
Collapsed	Clostridiales	Lachnospiraceae	Unclassified	0.23 [0;0.35]	-0.03(0.01)	0.0007	0.036	0.22(0.15)	0.145
190162	Clostridiales	Lachnospiraceae	Blautia	0.16 [0;0.29]	-0.16(0.04)	0.0005	0.033	0.95(0.68)	0.163
199710	Clostridiales	Christensenellaceae	Unclassified	0.14 [0;0.29]	0.18(0.05)	0.0011	0.046	-1.03(0.76)	0.175
195465	Clostridiales	Lachnospiraceae	Unclassified	0.02 [0;0.25]	-0.16(0.05)	0.0009	0.043	0.94(0.71)	0.184
289734	Clostridiales	Lachnospiraceae	Unclassified	0.29 [0;0.41]	-0.05(0.01)	0.0002	0.019	0.25(0.19)	0.190
177515	Clostridiales	Lachnospiraceae	Roseburia	0 [0;0.24]	-0.04(0.01)	0.0009	0.041	0.22(0.17)	0.208
295258	Clostridiales	Ruminococcaceae	Unclassified	0.15 [0;0.28]	0.09(0.02)	0.0005	0.033	-0.43(0.35)	0.170
175761	Clostridiales	Ruminococcaceae	Unclassified	0.15 [0;0.29]	0.26(0.08)	0.0011	0.046	-1.3(1.09)	0.223
181756	Clostridiales	Lachnospiraceae	Blautia	0 [0;0.0]	-0.22(0.06)	0.0003	0.026	1.04(0.87)	0.232
292735	Clostridiales	Lachnospiraceae	Blautia	0.03 [0;0.36]	-0.11(0.02)	0.0002	0.019	0.46(0.40)	0.253
197930	Clostridiales	Lachnospiraceae	Unclassified	0.01 [0;0.14]	-0.22(0.05)	0.0001	0.015	-0.85(0.79)	0.283
317677	Clostridiales	Ruminococcaceae	Unclassified	0.06 [0;0.2]	-0.23(0.06)	0.0003	0.022	-0.91(0.93)	0.330
181485	Clostridiales	Lachnospiraceae	Ruminococcus	0 [0;0.0]	-0.23(0.07)	0.0010	0.044	0.91(0.95)	0.341
194488	Clostridiales	Ruminococcaceae	Unclassified	0 [0;0.0]	0.22(0.05)	0.0002	0.021	0.69(0.76)	0.365
4446669	Clostridiales	Ruminococcaceae	Unclassified	0 [0;0.0]	0.21(0.06)	0.0006	0.035	-0.68(0.82)	0.408
186955	Clostridiales	Lachnospiraceae	Unclassified	0.09 [0;0.23]	-0.24(0.05)	2.69x10^-5^	0.009	0.62(0.78)	0.426
308544	Clostridiales	Ruminococcaceae	Unclassified	0.21 [0;0.38]	-0.10(0.03)	0.0011	0.047	0.33(0.43)	0.370
176604	Clostridiales	Lachnospiraceae	Unclassified	0.16 [0;0.29]	-0.12(0.03)	0.0002	0.019	0.32(0.43)	0.454
187917	Clostridiales	Ruminococcaceae	Unclassified	0.06 [0;0.26]	0.13(0.03)	0.0001	0.018	-0.31(0.46)	0.500
Collapsed	Clostridiales	Lachnospiraceae	Roseburia.uk	0.04 [0;0.63]	-0.04(0.01)	9.33x10^-6^	0.006	0.08(0.13)	0.512
199430	Clostridiales	Ruminococcaceae	Faecalibacterium prausnitzii	0.12 [0;0.25]	-0.17(0.05)	0.0007	0.037	0.43(0.69)	0.530
174288	Clostridiales	Ruminococcaceae	Unclassified	0.03 [0;0.16]	-0.16(0.04)	0.0005	0.032	-0.41(0.66)	0.531
1740283	Clostridiales	Lachnospiraceae	Roseburia	0.04 [0;0.17]	-0.21(0.05)	0.0001	0.017	0.46(0.77)	0.545
Collapsed	Clostridiales	Christensenellaceae	Unclassified	0.38 [0.21;0.5]	0.07(0.01)	3.54x10^-7^	0.001	-0.11(0.19)	0.550
4217963	Clostridiales	Lachnospiraceae	Unclassified	0.05 [0;0.18]	-0.29(0.06)	1.26x10^-5^	0.006	0.51(0.9)	0.572
Collapsed	Clostridiales	Christensenellaceae	Unclassified	0.38 [0.21;0.49]	0.07(0.01)	4.75x10^-5^	0.001	-0.10(0.19)	0.580
2298935	Clostridiales	Lachnospiraceae	Unclassified	0.01 [0;0.25]	-0.11(0.03)	0.0002	0.021	0.22(0.42)	0.604
4332082	Clostridiales	Lachnospiraceae	Roseburia	0.19 [0;0.31]	-0.21(0.05)	0.0003	0.026	0.40(0.79)	0.613
199761	Clostridiales	Ruminococcaceae	Unclassified	0.11 [0;0.24]	-0.08(0.02)	0.0006	0.033	0.16(0.32)	0.615
175509	Clostridiales	Lachnospiraceae	Blautia	0.01 [0;0.14]	-0.36(0.09)	0.0001	0.016	0.57(1.26)	0.647
3134492	Clostridiales	Lachnospiraceae	Unclassified	0.02 [0;0.24]	-0.06(0.01)	3.64x10^-5^	0.016	0.09(0.20)	0.651
231952	Clostridiales	Christensenellaceae	Unclassified	0.07 [0;0.21]	0.24(0.05)	1.58x10^-5^	0.007	-0.33(0.76)	0.620
334336	Clostridiales	Ruminococcaceae	Unclassified	0.08 [0;0.21]	-0.19(0.05)	0.0009	0.042	-0.30(0.75)	0.668
313593	Clostridiales	Lachnospiraceae	Roseburia	0 [0;0.11]	-0.11(0.02)	0.0003	0.021	-0.16(0.40)	0.690
3926480	Clostridiales	Lachnospiraceae	Roseburia	0.06 [0;0.19]	-0.06(0.01)	0.0007	0.037	-0.10(0.25)	0.693
187447	Clostridiales	Lachnospiraceae	Roseburia	0.02 [0;0.15]	-0.15(0.04)	0.0005	0.032	-0.22(0.63)	0.723
4094259	Clostridiales	Ruminococcaceae	Unclassified	0.18 [0;0.3]	-0.25(0.07)	0.0008	0.039	0.34(1.05)	0.739
190100	Clostridiales	Lachnospiraceae	Blautia	0.15 [0;0.28]	-0.16(0.05)	0.0012	0.047	0.18(0.71)	0.797
4468466	Clostridiales	Ruminococcaceae	Unclassified	0.34 [0.1;0.45]	0.04(0.01)	0.0001	0.015	0.03(0.15)	0.809
3265161	Clostridiales	Lachnospiraceae	Unclassified	0.18 [0;0.31]	-0.20(0.05)	4.89x10^-5^	0.012	-012(0.60)	0.848
4202174	Clostridiales	Clostridiaceae	Unclassified	0.15 [0;0.37]	0.07(0.02)	0.0008	0.040	-0.05(0.32)	0.864
Collapsed	Clostridiales	Ruminococcaceae	Oscillospira	0.13 [0;0.26]	0.09(0.02)	0.0006	0.034	0.06(0.37)	0.868
2250985	Clostridiales	Lachnospiraceae	Roseburia	0.10 [0;0.24]	-0.15(0.04)	0.0003	0.025	-0.08(0.55)	0.887
125624	Clostridiales	Lachnospiraceae	Unclassified	0.18 [0;0.15]	-0.05(0.01)	0.0001	0.015	0.02(0.21)	0.889
4370941	Clostridiales	Ruminococcaceae	Unclassified	0.28 [0.11;0.4]	0.19(0.04)	0.0001	0.015	-0.05(0.65)	0.929
3393191	Clostridiales	Lachnospiraceae	Roseburia	0.05 [0;0.18]	-0.14(0.04)	0.0007	0.038	-0.03(0.59)	0.957
730906	Clostridiales	Ruminococcaceae	Unclassified	0 [0;0.0]	-0.24(0.07)	0.0008	0.038	0.04(0.96)	0.960
300374	Clostridiales	Ruminococcaceae	Oscillospira	0.27 [0.11;0.3]	0.23(0.06)	0.0002	0.020	0.01(0.85)	0.983

* Heritability estimates comes from Goodrich JK et al. *Cell* 2014 [29].

**qval; is the significant threshold after apply false discovery rate (FDR <5%) adjustment. eGFR = estimated glomerular filtration rate.

Previous studies showed that Ruminococcaceae, Lachnospiraceae and Christensenellaceae families are associated with healthier phenotypes. Indeed, Ruminococcaceae and Lachnospiraceae families have been found to be inversely associated with inflammatory bowel disease and are considered butyrate producers [31,32]. Butyrate is a preferred energy source for colonic epithelial cells and is thought to play an important role in maintaining colonic health in humans. Additionally, Christensenellaceae has been recently described by our group to be inversely correlated with BMI in humans and in experimental murine models [29]. In our data, a higher abundance of members of these three families was associated with lower circulating levels of indoxyl-sulphate, p-cresyl-sulphate and phenylacetylglutamine and related to better renal function. In line with our findings, a reduction in the number of culturable anaerobic bacteria has been observed in CKD or on maintenance hemodialysis patients [33]. Our results suggest that CKD dysbiosis may start in earlier kidney function decline.

Heritability estimates for the three metabolites and the microbes identified are low/moderate heritability ranging from 0 to 0.38 (See Tables 2 and 3) suggesting that environmental factors have a major role in explaining the metabolite/microbe variation. Our heritability results are in line with those reported in non-twin population showing that metabolites derived from bacterial protein fermentation have low heritability [5].

Our study has some limitations. Firstly, the sample consists of predominantly healthy volunteer females with lower rate of diabetes and results may not be generalisable to males and to a population sample with greater prevalence of diabetes population. Moreover, estimates of GFR based on creatinine may underestimate renal function especially when GFR is >60 mL/min1.73m^2^. Cystain C has been proposed as an alternative marker of renal function that could aid to reduce the bias. However, Cystatin C is not measured on the TwinsUK cohort. However, we have tried to minimize the underestimation bias using the CKD-EPI formula.

The cross-sectional nature of our data does not allow us to draw conclusions as to whether the findings are causative of kidney function decline or merely correlated with it. Finally, our study does not provide absolute quantification of the metabolites, and future studies are needed to establish reference ranges for clinical use.

To our knowledge, this is first study combining metabolome and microbiome data in early renal function decline. Our results have the potential to identify at risk patients before the onset of advanced CKD. Also, they open new avenues to our understanding of the renal-gut-microbiota-metabolite axis, which could improve therapeutic strategies. As well as providing early markers of renal damage, the microbiome can be manipulated allowing early therapeutic possibilities for prevention.

## Concise Methods

### Study subjects

Study subjects were twins enrolled in the Twins UK registry, a national register of adult twins started in 1992. The registry consists of over 10,000 predominantly female monozygotic and dizygotic twins, 18–84 years old, comparable to the general population in terms of lifestyle characteristics. Healthy twins were recruited from all over the UK as volunteers by successive media campaigns without selecting for particular diseases or traits. The TwinsUK cohort represents one of the most detailed omics and phenotypic resource in the word [34].

Data relevant to the present study include, BMI (body weight in kilograms divided by the square of height in square meters), type 2 Diabetes (t2D) (defined if fasting glucose ≥7 mmol/L or physician’s letter confirming diagnosis). Renal parameters include estimated glomerular filtration rate (eGFR) calculated from standard creatinine using the CKD-EPI equation [35].

Dietary scores were obtain from food frequency questionnaires (FFQ) summarizing fruit and vegetable intake, alcohol intake, meat intake, hypo-caloric dieting and a ‘‘traditional English” diet as previously describe [25,26]. These five dietary scores are principal component analysis generated scores. As such they are independent variables standardized to have mean of zero and a SD of one in the whole TwinsUK study population. Each dietary pattern should be considered as the representative of a particular food pattern intake

Individuals were requested to complete a questionnaire regarding antibiotics used within the month previous faecal sample collection.

St. Thomas’ Research Ethics Committee approved the study (EC96/439 TwinsUK) and all participants provided informed written consent.

### Measurement of Metabolites

Non-targeted gas chromatography/mass spectrometry-based profiling was performed fasting plasma samples from participants in the TwinsUK cohort, using the Metabolon platform, as described previously [36,37]. Briefly, the Metabolon platform integrates the chemical analysis, including identification and relative quantification, data reduction, and quality assurance components of the process. This integrated platform enables the high-throughput collection and relative quantitative analysis of analytical data and identified a large number and broad spectrum molecules with a high degree of confidence. We inverse-normalised the metabolomics data and excluded metabolic traits with >20% missing values.

### Microbiota analysis

Faecal samples were obtained from adult twin volunteers in the TwinsUK cohort. Faecal sample collection and 16S rRNA sequencing are described in depth previously in this sample (Goodrich et al) [29]. Briefly, the V4 region of the 16S rRNA gene was amplified and sequenced on Illumina MiSeq. Quality filtering and analysis of the sequence data with QIIME 1.7.0, was followed by closed-reference OTU picking to select OTUs at 97% sequence identity against the Greengenes May 2013 database as previously reported [38]. OTUs were adjusted for age, gender, shipment, number of sequences per sample and sequencing run. Collapsed taxonomic bins were created by combining OTUs of the same taxonomic designation into one variable. In total we used 768 OTUs and 141 collapsed taxonomies.

### Statistical analysis

Statistical analysis was carried out using Stata version 12 and R version 3.1.2 (package LME4). Association analyses between eGFR and metabolites or microbiota profiles were performed using random intercept linear regressions adjusting by age, sex, BMI, diabetes, experiment batch and family relatedness. Linear Mixed Effects Regression (LMER) was used to test the association between the microbiota and metabolites. Family structure and twin zygosity were accounted for as random effects and the microbe was the predictor variable. Multiple testing correction for the microbiota analysis was performed via false discovery rate (FDR<5%).
